# Nashville Medical Recorder

**Published:** 1858-11

**Authors:** 


*Nashville Medical Recorder.*—We have received the first number of a new Journal started at Nashville, which is a union of the Memphis Medical Recorder and Southern Journal of the Medical and Physical Sciences, edited by Drs. Daniel F. Wright and R. G. Curry, late editors of those Journals. These gentlemen now hold professorships in the Shelby Medical College of Nashville, and the Journal will be probably devoted to the interests of that school. The first number presents a good appearance, and the reputation of its editors as journalists leaves no doubt as to its future success. We have unfortunately mislaid the first number, and depend upon the kindness of the editors for another copy.

